# A Clinical Challenge in the Emergency Department: A Case of Klebsiella Infective Endocarditis Presenting With Splenic Abscess

**DOI:** 10.7759/cureus.10577

**Published:** 2020-09-21

**Authors:** Nicolas Ulloa, Jessica M Cook, Shaun Smithson

**Affiliations:** 1 Emergency Medicine, Aventura Hospital and Medical Center, Aventura, USA; 2 Cardiology, Aventura Hospital and Medical Center, Aventura, USA

**Keywords:** endocarditis, splenic abscess, emergency medicine, critical care, cardiology

## Abstract

Infective endocarditis (IE) is a serious bacterial infection of the endocardium and/or heart valves that carries considerable morbidity and mortality. Often presenting with very non-specific symptoms, this disease presents many challenges to the emergency medicine practitioner.

A 47-year-old male with no pertinent medical history presented to the emergency department complaining of shortness of breath. He stated that his symptoms had been persistent for the last three weeks and were associated with malaise and fatigue. CT of the abdomen/pelvis with IV contrast revealed a 7-cm hypodensity of the spleen concerning for abscess versus infarct. He denied any trauma or IV drug use. Follow-up ultrasound was ordered, which characterized the hypodensity as a splenic abscess. An echocardiogram was recommended for possible IE, and cardiology was consulted. The transthoracic echocardiogram was performed on hospital day 2, which showed minimal mitral valve thickening with mild mitral regurgitation. The interventional radiology (IR) service was consulted for the splenic abscess in order to perform CT-guided drainage. An IR drain was successfully placed on hospital day 3. On the same day, blood cultures grew Klebsiella pneumoniae. On hospital day 5, that patient was transferred to the ICU for possible empyema formation with signs of respiratory distress. The patient underwent CT of the chest that showed the development of a left-sided effusion. The patient had also been persistently tachycardic and febrile, with high leukocytosis since admission and worsening respiratory status. Transesophageal echocardiogram (TEE) was scheduled but put on hold due to worsening respiratory status. Repeat TEE was scheduled five days later, which showed mitral regurgitation and increased size of the vegetation despite antibiotic therapy. Two days later, he was scheduled for mitral valve repair.

When reviewing our case, the patient had both common and uncommon aspects of splenic abscess or IE. First, despite having respiratory symptoms for two weeks, the primary reason he came to the hospital was due to the new onset of fevers. He was febrile, tachycardic, and with significant leukocytosis. He continued to have fevers despite antibiotic therapy and IR drainage of the abscess. With no history of IV drug use history, negative transthoracic echocardiography, lack of immunocompromising condition, and blood cultures with gram-negative rods, IE became less likely of a diagnosis.

Establishing the diagnosis of IE proved to be exceptionally complicated, especially in the setting of a COVID-19 pandemic. The most notable challenge was having a high index of suspicion despite any risk factors. The patient was a previously healthy 47-year-old male with no medical problems.

IE continues to be a clinical challenge for physicians, especially in the emergency department, due to the lack of diagnostic criteria such as positive blood cultures or vegetations visualized on echocardiographic studies. IE has a wide gamut of presentations with different levels of acuity. Diagnosis is more straightforward when patients present with obvious risk factors, but, in many cases, such as this one, those risk factors may be absent. A high index of suspicion is required, especially in patients with additional findings such as splenic abscess, embolic phenomenon, focal neurologic deficit, mycotic aneurysm, decompensated heart failure, new murmurs, or pleural effusions.

## Introduction

Infective endocarditis (IE) is a serious bacterial infection of the endocardium and/or heart valves that carries considerable morbidity and mortality. Often presenting with non-specific symptoms, such as fever, chills, fatigue, and shortness of breath, this disease presents several challenges to the physician in the emergency department (ED). Patients may lack well-known risk factors such as intravenous drug use, previous history of IE, and prosthetic heart valves, which can make it more difficult to consider in the differential diagnosis, especially during the influenza season and novel coronavirus disease 2019 (COVID-19) pandemic in which there is an abundance of patients presenting with flu-like illness. It is important for the emergency medicine (EM) clinician to be aware of the multiple manifestations of IE on history and examination, especially in the absence of such risk factors. The foundation of establishing a diagnosis requires blood cultures and definitive echocardiographic findings, neither of which is considered during the patient’s time in the ED. Early consideration, recognition, and treatment of IE are crucial for improved outcomes. Obtaining timely blood cultures and administering appropriate empiric antibiotics in the ED coupled with early transthoracic/transesophageal echocardiogram (TTE/TEE) are essential to guiding therapy once the patient is admitted to the hospital.

## Case presentation

**Figure 1:**
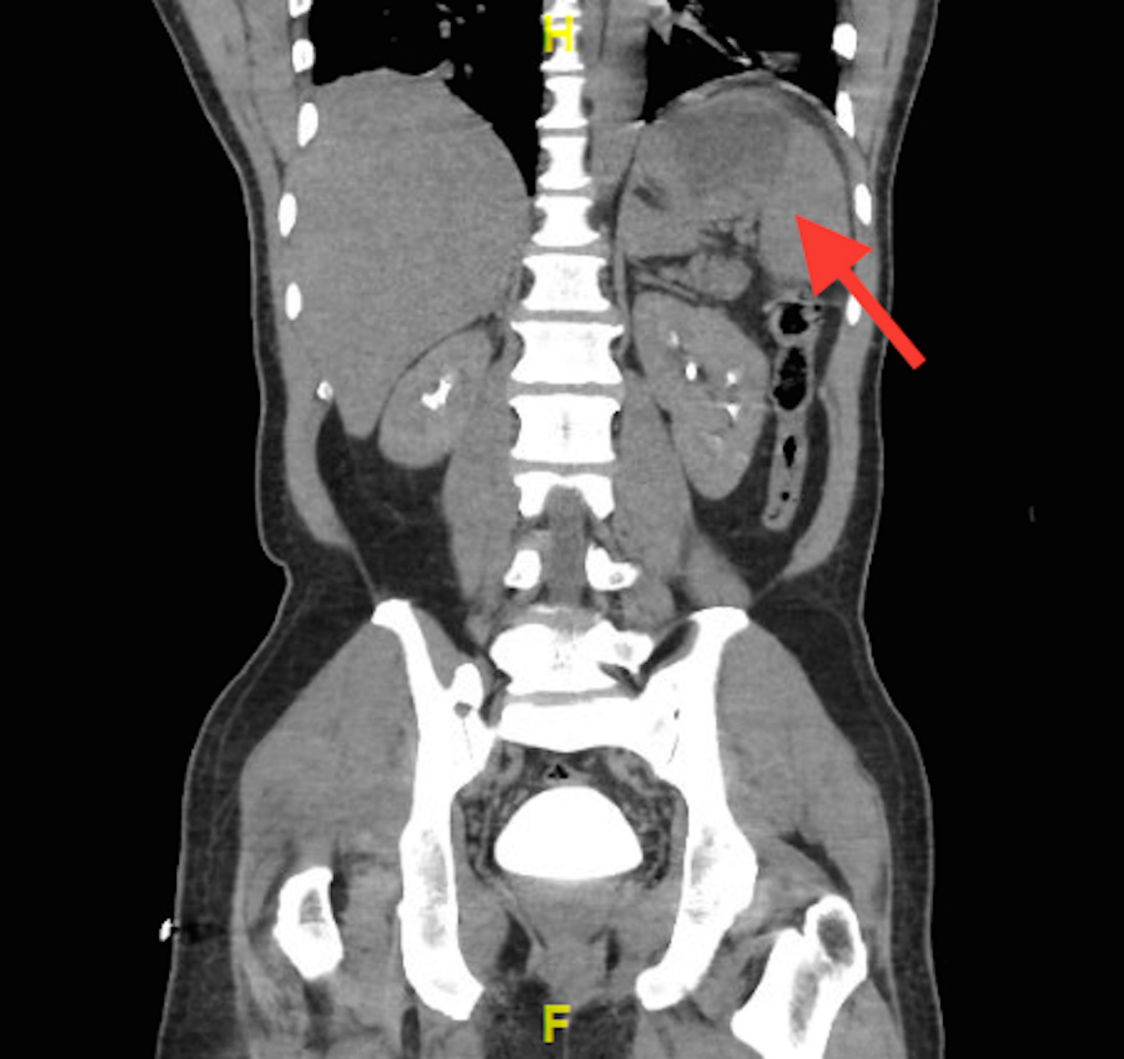
CT of the abdomen/pelvis coronal view demonstrating hypodensity in the spleen, read as infarct versus abscess

A 47-year-old male with no pertinent medical history presented to the ED complaining of shortness of breath. He stated that his symptoms had been persistent for the last three weeks and he also reporting malaise and fatigue. He was seen at a different hospital previously for the same symptoms and was discharged home with an albuterol inhaler, with no improvement. A few days prior to arrival, his symptoms worsened and he started to experience subjective fevers, which led to his second ED visit. While in the ED, his vitals showed a low-grade fever of 99.2°F, tachycardia ranging from 102-144, a normal blood pressure of 145/78, and oxygen saturation of 98%. The ED team pursued a diagnostic workup for suspected COVID-19. Physical examination was notable for an ill-appearing and diaphoretic male. Cardiac examination revealed tachycardia without any obvious murmurs. Pulmonary examination was notable for tachypnea but otherwise clear to auscultation. The abdomen was soft and non-tender. His lab workup was notable for a leukocytosis of 29.1 x 109/L (normal range: 3.6-11.0 x 109/L) and elevation in his inflammatory markers. He had an initial C-reactive protein of 18.7 mg/dL (normal range: 0-0.1 mg/dL), ESR of 65 mm/hr (normal range: 0-10 mm/hr), lactate dehydrogenase of 267 units/L (normal range: 84-246 units/L), and D-dimer of 2,361 ng/mL (normal range: 0-316 ng/mL), which are all elevated. His lactic acid was within the normal range. Of note, his troponin was moderately elevated at 0.367 ng/dL (normal range: 0.000-0.043 ng/dL). The chest X-ray was unremarkable and the computed tomography (CT) angiogram of the chest was negative for any infectious etiology, pulmonary embolism, or dissection. CT of the abdomen/pelvis with IV contrast revealed a 7-cm hypodensity within the spleen, concerning for abscess versus infarct (Figure 1). He denied any trauma or IV drug use. Follow-up ultrasound was ordered which characterized the hypodensity as a splenic abscess. The patient received a “sepsis bundle”, which included the administration of IV fluids, ceftriaxone, and azithromycin to cover empirically for COVID co-infection; blood cultures were drawn in the ED and he was subsequently admitted to the “COVID rule-out” medical floor of the hospital.

**Figure 2:**
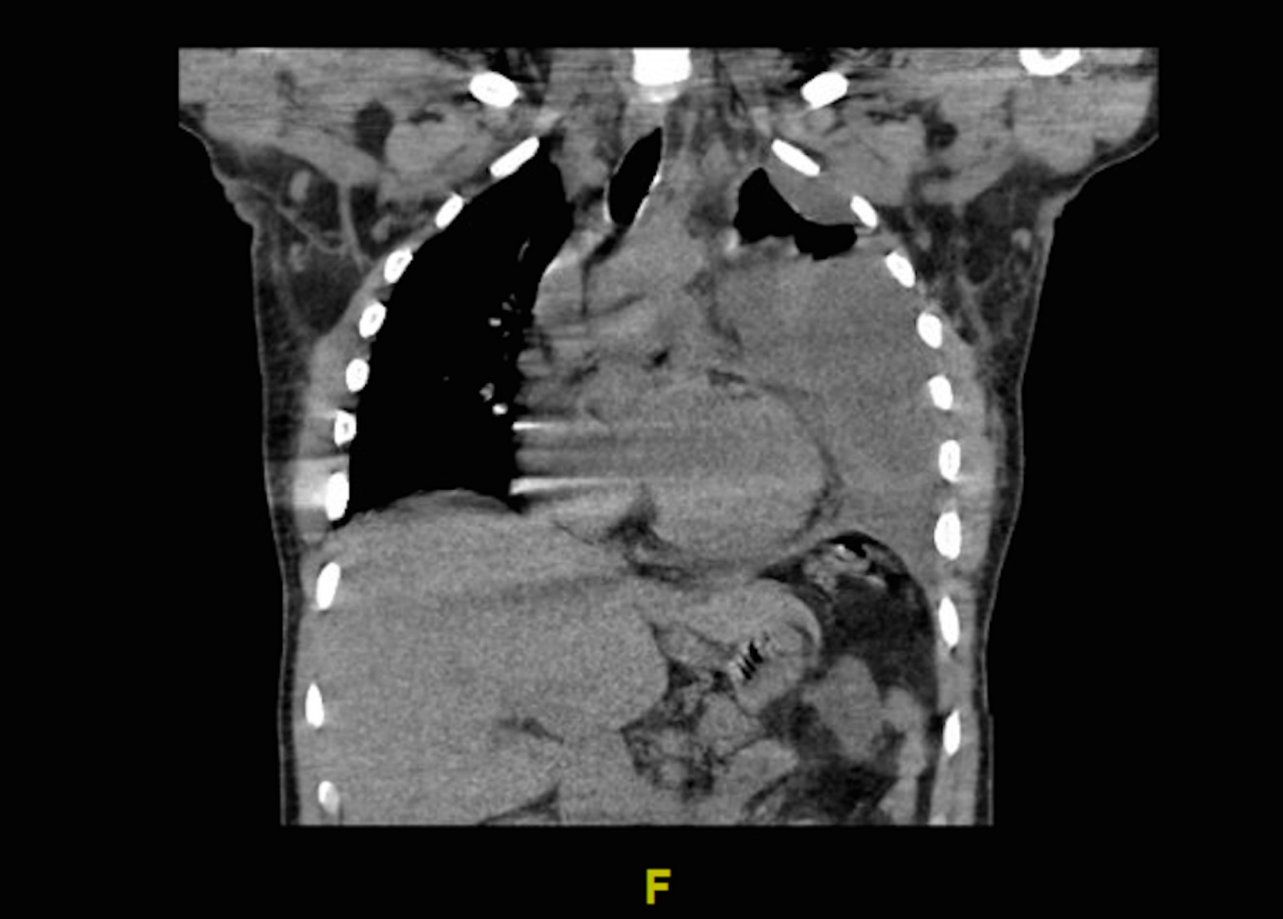
CT of the chest coronal view demonstrating multiloculated left-sided pleural effusion, which developed after IR drainage of the spleen, likely secondary to the splenic abscess or infective endocarditis IR, interventional radiology

The patient tested negative for COVID-19 within a few hours. The infectious disease service was consulted and adjusted the antibiotic regimen to vancomycin and piperacillin-tazobactam. An echocardiogram was recommended to rule out IE and the cardiology service was consulted. The TTE was performed on hospital day 2, which showed minimal mitral valve thickening with mild mitral regurgitation. The interventional radiology (IR) service was consulted for the splenic abscess with plans to perform CT-guided drainage. An IR drain was successfully placed on hospital day 3. On the same day, blood cultures grew *Klebsiella pneumoniae*. On hospital day 5, the patient was transferred to the ICU due to respiratory distress and suspected new empyema formation. A CT of the chest was ordered at that time, which showed the development of a left-sided pleural effusion, as seen in Figure 2. The patient had also been persistently tachycardic and febrile, with high leukocytosis and gradually worsening respiratory distress since admission. TEE was scheduled but was postponed due to worsening respiratory status. At this point, the cardiothoracic surgery service was consulted. They scheduled the patient for left-sided lung decortication the following day. While still intubated from the procedure, the patient was scheduled for TEE the following morning, which revealed IE of the mitral valve. Findings were discussed with cardiothoracic surgery service that initially recommended medical management. Repeat TEE was scheduled five days later, which showed mitral regurgitation and increased size of the mitral vegetation despite aggressive antibiotic therapy. Two days later, the patient was scheduled for mitral valve repair by CT surgery. Postoperatively, the patient's tachycardia resolved, leukocytosis improved, and his temperature normalized. He continued to improve during his hospital stay and was discharged 10 days later without complications.

## Discussion

Splenic abscess

Splenic abscess is a fairly uncommon disease. Some data suggest it has an incidence as low as 0.2-0.7% [1]. There are a variety of etiologies in which a patient can develop a splenic abscess, but most commonly, after trauma, and in patients who are immunocompromised, it develops secondary to disseminated infection [1]. It is important to note that up to 5% of IE cases are complicated by splenic abscess formation [2]. A splenic abscess most commonly presents with various non-specific symptoms such as fever, abdominal pain, and chills; this can make the diagnosis difficult based on clinical presentation alone. It should also be considered in patients who have recurrent or persistent fevers despite adequate antibiotic regimen. Fortunately, the increasing use and accuracy of ultrasonography and CT have aided in confirming the diagnosis.

A review of the literature helps illuminate some of the more common presentations of splenic abscess. Specifically, fever has been noted by some sources to be present in as high as 92.6% of patients [1,3]. Additionally, 55.6-66.7% had abdominal pain and 61.1-89% had a leukocytosis [1,3]. Of note, some data suggest that up to 88% of patients had left-sided pleural effusions that were noted on chest X-ray [1]. In terms of microbiology, sources have mentioned that *Streptococcus viridans*, *Staphylococcus aureus*, and *Klebsiella Pneumoniae* tend to frequently be identified as the causative agents [1,2]. Mortality is consistently cited at 14-16% [1,3]. Currently, there is no gold standard approach to the management of splenic abscesses. The three current modalities of treatment are IV antibiotics alone, percutaneous drainage, and splenectomy. The literature shows they have similar survival rates, and the recommendation is that treatment should vary on a case by case basis [3].

When reviewing our case, the patient had both classic and subtle presentations of splenic abscess. Despite having respiratory symptoms for two weeks, the primary reason he presented to the hospital was due to the onset of new fevers. He was febrile and tachycardic, with a significant leukocytosis. He continued to have fevers despite antibiotic therapy and IR drainage of the abscess. After the development of a left-sided pleural effusion and empyema formation, which has been well documented to co-exist with splenic abscesses, he required decortication of the left lung. Despite these interventions, the patient remained febrile and tachycardic until he had the infected valve repaired. Blood cultures were positive for Klebsiella pneumoniae, which is uncommon but has been noted in the literature when reviewing splenic abscesses. Despite this, he did not fit into any high-risk population. He denied any trauma preceding his symptoms. Furthermore, he lacked any immunocompromising conditions such as HIV, end-stage renal disease, or diabetes. The patient was a previously healthy 47-year-old male with no hospitalizations prior to this one. He adamantly denied any drug use, which was confirmed by a negative urine drug screen. The absence of IV drug use history, negative TTE, lack of immunocompromising condition, and blood cultures that grew gram-negative rods all pointed away from IE as the primary etiology.

Infective endocarditis 

IE is an infection of the endothelial layer of the heart and can disseminate rapidly. This disease continues to be diagnostically challenging for EM physicians to diagnose primarily due to a variety of presentations, breadth of acuity, and often absence of identifiable risk factors.

**Figure 3:**
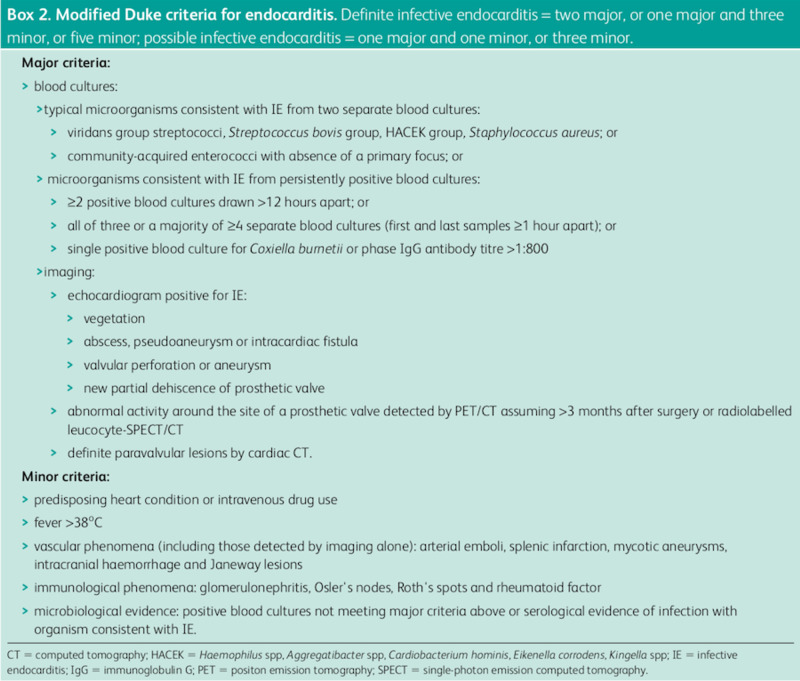
Modified Duke's criteria [4]

The modified Duke’s criteria, as shown in Figure 3, are the standard for which clinicians diagnose IE. Recent literature updates demonstrate that these criteria have an 80% sensitivity in establishing a diagnosis [4]. Unfortunately, very few aspects can be readily applied in the ED. Positive blood cultures and echocardiographic findings are the hallmark tests for diagnosing IE. While non-invasive bedside TTE is somewhat sensitive if the vegetation is large, the blood culture results are not available when the patient is presenting acutely ill in the first few hours of their ED hospitalization.

An article was written by Delaney regarding IE in the ED in which she established guidelines to increase detection and determine who may benefit from hospitalization and further workup, specifically, febrile elderly patients in the absence of a clear source, patients with prolonged fever of more than two weeks without clear etiology, patients with evidence of vasculitis or embolization with a new murmur or change in old murmur, patients with fever plus known structural heart disease or recent instrumentation without a clear source, patients with fever and with cardiac prosthesis or any duration of malaise, vasculitis, or new murmur, and, finally, any febrile IV drug users [5].

Given the increasing prevalence of structural heart disease that is associated with aging, elderly patients with fever lasting more than two weeks should undergo workup that includes IE. Additionally, they are more susceptible to bacterial illnesses. An individual with fevers longer than two weeks is unlikely to have a benign etiology and should be further evaluated. Signs of vasculitis, embolization, and new murmurs should lead to an increased index of suspicion as they can be signs of acute pathology and disseminated infection. Predisposing conditions such as structural heart disease, prosthetic heart valves, and IV drug use are significant risk factors for IE, and anyone with an associated fever without a clear source should be further evaluated [5]. Although quite dated, the article illustrates significant key features that can aid the EM physician in considering IE. Of note, other significant risk factors include previous diagnosis of IE, immunocompromised state, indwelling lines for venous access, and chronic kidney disease [4].

Echocardiography remains a critical element in diagnosing IE. TTE tends to be the first image modality due to its convenience and non-invasiveness. Despite this, the sensitivity of TTE in diagnosing IE is merely 71% [6]. There is a direct correlation between the size of the vegetation and the detection on TTE. The literature demonstrates that TTE has a sensitivity of 25%, 70%, and 84% for vegetations < 5 mm, 6-10 mm, and >10 mm, respectively [6]. Although there is a lack of literature comparing POCUS (point-of-care ultrasound) in the ED to a gold standard, it is reasonable to assume that sensitivities may be more variable than the standard of care TTE, depending on the EM providers proficiency with bedside ultrasound. Any patient with a high degree of suspicion for IE and a negative TTE should be followed up with a TEE when possible, as they have a sensitivity of 90% [4].

IE is associated with significant morbidity and mortality, making early diagnosis with appropriate therapy paramount. Some studies have noted a 30-day mortality of 30% [4]. A Korean study compared the difference between early surgical intervention and conventional medical treatment, with primary outcomes being hospital death or embolic events within six weeks. The research showed a statistically significant difference between the two groups respectively (3% vs 23%) [7]. The main difference is observed in the reduced embolic rates noted in the early surgery group [7]. The study reinforces the importance of early intervention, which requires a timely diagnosis.

When reviewing our case, establishing the diagnosis of IE proved to be exceptionally complicated, especially in the setting of the COVID-19 pandemic. The most notable challenge was having a high index of suspicion for IE despite the lack of risk factors. The patient was a previously healthy 47-year-old male with no medical problems. He had no immunocompromising conditions, no congenital/structural heart defects, and normal dentition. Of note, he adamantly denied drug use. On examination, he was ill-appearing, febrile, and tachycardic, with laboratories significant for high leukocytosis, elevation in inflammatory markers, and mild troponin elevation, which are non-specific. The key feature of the disease process was that CT of the abdomen/pelvis revealed an acute splenic abscess. In the absence of other risk factors for abscess, disseminated disease from IE had to be considered. Even after a non-diagnostic TTE, a follow-up TEE would have ideally been performed next. Unfortunately, the patient’s respiratory status deteriorated after the development of a left-sided empyema, which delayed the procedure, diagnosis, and, ultimately, the treatment.

The management of IE in the setting of an associated splenic abscess requires certain considerations. For example, some suggest splenectomy/drainage should take place before valve repair to prevent secondary infection [1]. Depending on the stability of the patient's condition, there is literature to suggest that splenectomy/drainage can occur simultaneously with valve repair as a one-stage process [1]. Our patient’s course was complicated by ongoing fevers and tachycardia and the development of a left-sided empyema even after drainage of the splenic abscess, which delayed TEE until after his lung decortication. His clinical picture began to improve after his mitral valve was repaired, and the remainder of his hospital course was uneventful.

## Conclusions

IE continues to be a clinical diagnostic challenge for physicians, especially in the ED, due to the lack of diagnostic criteria such as immediate positive blood cultures or ease of visualized vegetations on bedside echocardiographic studies. IE has a wide gamut of presentations with different levels of acuity. Diagnosis is more straightforward when patients present with obvious risk factors, but, in many cases, such as this one, those risk factors may be absent. A high index of suspicion is required, especially in patients with additional findings such as splenic abscess, embolic phenomenon, focal neurologic deficit, mycotic aneurysm, decompensated heart failure, new murmurs, or pleural effusions. Additionally, a contemporary scoring system/decision-making tool should be proposed and further studied to aid clinicians in earlier diagnosis, especially in the ED. IE carries significant mortality and would justify an aggressive push for performing TEE early in the hospital course. Protocols should be established to facilitate this process, aiding in prompt diagnosis, appropriate therapy, and improved patient outcomes.
